# Two new soil-inhabiting fungi, *Malbranchea cavernicola* (Onygenales, Malbrancheaceae) and *Talaromyces pangmaphaensis* (Eurotiales, Trichocomaceae), from a cave in northern Thailand

**DOI:** 10.3897/mycokeys.131.188256

**Published:** 2026-04-22

**Authors:** Gui-Qing Zhang, Nakarin Suwannarach, Terd Disayathanoowat, Rasmi Shoocongdej, Varis Domethong, Dong-Qin Dai, Rui-Meng Liu, Nalin N. Wijayawardene, Jaturong Kumla

**Affiliations:** 1 Department of Biology, Faculty of Science, Chiang Mai University, Chiang Mai 50200, Thailand College of Biology and Food Engineering, Qujing Normal University Qujing China; 2 Center of Excellence in Microbial Diversity and Sustainable Utilization, Chiang Mai University, Chiang Mai 50200, Thailand Department of Archeology, Faculty of Archeology, Silpakorn University Bangkok Thailand; 3 Center for Yunnan Plateau Biological Resources Protection and Utilization & Yunnan International Joint Laboratory of Fungal Sustainable Utilization in South and Southeast Asia, College of Biology and Food Engineering, Qujing Normal University, Qujing 655099, China Faculty of Science, Chiang Mai University Chiang Mai Thailand; 4 Office of Research Administration, Chiang Mai University, Chiang Mai 50200, Thailand Center of Excellence in Microbial Diversity and Sustainable Utilization, Chiang Mai University Chiang Mai Thailand; 5 The Conservation of Ancient Log Coffins of Phi Man Long Long Rak Cave, Mae Hong Son Province Project, Princess Maha Chakri Sirindhorn Anthropological Centre, 20, Boromrachachonnani Road, Taling Chan, Bangkok 10170, Thailand Office of Research Administration, Chiang Mai University Chiang Mai Thailand; 6 Department of Archeology, Faculty of Archeology, Silpakorn University, Bangkok 10200, Thailand The Conservation of Ancient Log Coffins of Phi Man Long Long Rak Cave, Mae Hong Son Province Project, Princess Maha Chakri Sirindhorn Anthropological Centre Bangkok Thailand; 7 Department of Bioprocess Technology, Faculty of Technology, Rajarata University of Sri Lanka, Mihintale 50300, Sri Lanka Faculty of Technology, Rajarata University of Sri Lanka Mihintale Sri Lanka

**Keywords:** Cave fungi, multi-locus phylogeny, new taxa, taxonomy, tropical area

## Abstract

Caves are globally recognised as reservoirs of high fungal diversity, with cave soils harbouring many previously underexplored taxa. Fungal research in Thailand has been conducted for more than two decades; however, studies focusing on fungal diversity in cave environments remain limited. In the present study, six fungal strains were isolated from soil samples collected from the prehistoric Phi Man Long Long Rak Cave located in Pang Mapha District, Mae Hong Son Province, northern Thailand. These strains were identified, based on morphological characteristics and multi-locus phylogenetic analyses using sequences of the internal transcribed spacer (ITS), the nuclear ribosomal large subunit (LSU), β-tubulin (*BenA*), calmodulin (*CaM*) and the RNA polymerase II second largest subunit (*RPB*2). Four strains representing two new species, *Malbranchea cavernicola* and *Talaromyces pangmaphaensis*, are described herein. In addition, two strains identified as *Malbranchea gymnoascoides* are reported for the first time from soil and represent a new geographical record in Thailand. Full descriptions, illustrations and a phylogenetic tree showing the phylogenetic positions of the cave fungi identified in this study are provided.

## Introduction

Caves are characterised by darkness, high humidity, consistently low temperatures, oligotrophic conditions and minimal photosynthetic activity (Joshi and Chettri 2019). The diverse substrates present in cave environments, including cave air, cave walls, the fur/skin of living bats, organic litter (e.g. animal faeces, carcasses, bat guano and plant debris), rocks, soils/sediments and water, support a high diversity of fungal species (Gabriel and Northup 2013). Cave fungi are globally distributed and well adapted to subterranean environments (Zhang et al. 2018; Savković et al. 2025). Zhang et al. (2021) provided a list of 1923 cave fungal species belonging to 720 genera documented in caves and mines worldwide by 2021. Several previous studies have demonstrated that cave soils and cave air harbour numerous potential sources of novel fungal species (Vanderwolf et al. 2013; Zhang et al. 2017, 2021; Pereira et al. 2022; Preedanon et al. 2023; Ogórek et al. 2024). Thailand harbours extensive karst landscapes and limestone cave systems, particularly in the northern region (Dunkley et al. 2017). Despite the country’s recognised status as a biodiversity hotspot, fungal diversity in Thai caves remains poorly explored. To date, only a limited number of studies have investigated cave-associated fungi in Thailand (Nuankaew et al. 2022; Preedanon et al. 2023; Suetrong et al. 2023).

*Malbranchea* Sacc. was introduced by Saccardo in 1882, with *M. pulchella* Sacc. & Penz. as the type species, which was isolated from wet cardboard in France (Saccardo 1882). Historically, *Malbranchea* was placed within the family Onygenaceae Berk., order Onygenales Cif. ex Benny & Kimbr. (Currah 1985; Sarrocco et al. 2015; Rodríguez-Andrade et al. 2021). However, the integrative taxonomic studies combining morphological comparisons and multi-locus phylogenetic analyses (ITS, LSU, *BenA*, *TEF*1, *TEF*3, *RPB*6, *RPB*1 and *RPB*2) have led to its reclassification into the family Malbrancheaceae Kandemir & de Hoog in Onygenales (Kandemir et al. 2022). The anamorph of *Malbranchea* is characterised morphologically by hyaline to lightly pigmented hyphae and the production of arthroconidia formed by hyphal fragmentation, while the teleomorph is characterised by ascomata formed from an anastomosing network of orange to brown, ornamented or unornamented, thick-walled hyphae, bearing appendages and/or spine projections, with inflated asci that produce eight globose to oblate ascospores (Rodríguez-Andrade et al. 2021). Members of *Malbranchea* are keratinolytic and saprobic fungi commonly associated with soil, decaying organic matter, animal dung and keratin-rich substrates (e.g. hair, feathers and skin debris), and some species have been reported in association with animal and human diseases (Emmons 1954; Orr and Kuehn 1963; Sigler et al. 2002; Hubka et al. 2013). Currently, 40 accepted species are listed in Species Fungorum (accessed on 15 March 2026), excluding synonymous names.

The genus *Talaromyces* C.R. Benj. was established with *T. vermiculatus* (P.A. Dang.) C.R. Benj. (current name: *T. flavus*) as the type species (Benjamin 1995). Species of *Talaromyces* are widely distributed across various substrates and environments, including soils and water (freshwater and marine), air and materials associated with plants, animals or humans (Zang et al. 2023). Members of the genus exhibit considerable morphological diversity, typically producing biverticillate penicilli with smooth- to rough-walled conidia and are readily distinguished from closely-related genera by a combination of morphological and molecular characters (Pyrri et al. 2021). Recent advances in multi-locus phylogenetics (*BenA*, *CaM*, *RPB*2 and ITS) and integrative morphology have greatly refined the systematics of *Talaromyces*, leading to the recognition of numerous new species and the reorganisation of infrageneric classification into eight sections: sect. *Bacillispori*, *Helici*, *Islandici*, *Purpurei*, *Subinflati*, *Talaromyces*, *Tenues* and *Trachyspermi* (Yilmaz et al. 2014; Sun et al. 2020). More than 249 epithets of *Talaromyces* are currently listed in Species Fungorum (accessed on 19 March 2026). Currently, 49 species are recognised in *Talaromyces* sect. *Trachyspermi*, which has been restructured into five series: ser. *Diversi* (six species), ser. *Erythromelles* (13 species), ser. *Miniolutei* (10 species), ser. *Resinarum* (six species) and ser. *Trachyspermi* (14 species) (Peng et al. 2025).

During an investigation of cave fungi in northern Thailand in 2024, six fungal strains were isolated from soil samples collected from Phi Man Long Long Rak Cave, Pang Mapha District, Mae Hong Son Province, northern Thailand. This cave represents an important archaeological site and the only systematically investigated location of log coffins in Southeast Asia (Shoocongdej 2014; Samrit and Shoocongdej 2024). However, its fungal diversity has not yet been explored. Therefore, the objective of this study is to characterise and accurately identify these six fungal strains. Based on morphological characteristics and molecular evidence, four strains were assigned to *Malbranchea*, including one new species described here as *M. cavernicola* and two strains identified as *M. gymnoascoides*. The remaining two strains belonged to *Talaromyces* sect. *Trachyspermi* ser. *Miniolutei* and are introduced as a new species, *Talaromyces pangmaphaensis*. The taxa obtained in this study are illustrated and described in detail, based on morphological characteristics and multi-locus phylogenetic analyses. Furthermore, species delimitation and confirmation of the novel taxa were supported by the genealogical concordance phylogenetic species recognition (GCPSR) criteria using pairwise homoplasy index (PHI) test.

## Material and methods

### Sample collection and fungal isolation

Soil samples were collected from Phi Man Long Long Rak Cave, Pang Mapha District, Mae Hong Son Province, Thailand, in 2024 following the method described by Zhang et al. (2017). The soil samples were placed in individual zip-lock plastic bags, kept in an ice box and transported to the microbiology laboratory at the Center of Excellence in Microbial Diversity and Sustainable Utilization, Chiang Mai University. Fungal isolation was carried out using a modified dilution plate method based on Zhang et al. (2021). One gram of soil sample was suspended in 9 ml of 0.85% w/v sodium chloride (NaCl) solution in a test tube and thoroughly mixed using a vortex mixer. The suspension was then serially diluted in 10-fold steps up to 10^–3^. One hundred microlitres from each dilution were spread on to Dichloran Rose-Bengal chloramphenicol (DRBC) agar. The plates were incubated at 25 °C for one week and monitored every 48 hours for fungal growth. Hyphal tips from individual colonies were transferred to a new potato dextrose agar (PDA) supplemented with 50 µg/ml chloramphenicol and incubated at 25 °C in darkness to obtain pure cultures. Pure fungal strains were deposited in the culture collection of the Sustainable Development of Biological Resources, Faculty of Science, Chiang Mai University (SDBR-CMU), Chiang Mai Province, Thailand. Additionally, the holotype (preserved in a metabolically inactive state) was deposited in the Chiang Mai University Biology Department’s Herbarium (CMUB). Novel taxa have been registered at the MycoBank following Article F.5, International Code of Nomenclature for algae, fungi and plants (Shenzhen Code).

### Morphology observation

Fungal colonies of *Malbranchea* strains were cultured on oatmeal agar (OA), potato carrot agar (PCA), PDA and phytone yeast extract agar (PYEA) and incubated at 25 °C in the dark for 4 weeks, following Torres-García et al. (2023). *Talaromyces* strains were cultured on five different media: czapek yeast extract agar (CYA), OA, PDA, malt extract agar (MEA), yeast extract sucrose agar (YESA) and incubated at 25 °C in the dark for 7 days, following Sun et al. (2020) and Peng et al. (2025). Colony diameter, morphological characteristics and pigment production were investigated and recorded. Colour names and codes follow Kornerup and Wanscher (1967). Micromorphological features were examined using a light microscope (Nikon Eclipse Ni-U, Japan). Size data of microscopic structures were based on at least 30 measurements of each structure using the Tarosoft® Image Framework software and further processed using Adobe Photoshop CC 2018 (Adobe Systems, USA).

### DNA extraction, polymerase chain reaction and sequencing

Fresh fungal colonies on PDA plates were incubated at 25 °C in the dark for five days and mycelia were scraped off with a sterile scalpel blade. Genomic DNA was extracted according to the instructions of the DNA Extraction Mini Kit (FAVORGEN, China). The ITS, LSU, *BenA*, *CaM* and *RPB*2 loci were amplified using the primer pairs ITS5/ITS4 (White et al. 1990), LR0R/LR5 (Vilgalys and Hester 1990), Bt2a/Bt2b (Glass and Donaldson 1995), CMD5/CMD6 (Hong et al. 2006) and fRPB2-5f/fRPB2-7cr (Liu et al. 1999), respectively. Polymerase chain reaction (PCR) amplifications were performed in separate reactions in a total volume of 20 µl containing 1 µl of genomic DNA template, 1 µl of each forward and reverse primer, 10 µl of 2× Quick Taq^TM^ HS Dye-Mix (TOYOBO, Japan) and 7 µl of sterile deionised water. PCR amplification of ITS for both *Malbranchea* and *Talaromyces* and LSU, *BenA* and *RPB*2 for *Malbranchea*, was performed with an initial denaturation at 95 °C for 5 min, followed by 35 cycles of denaturation at 95 °C for 30 s, annealing at 56 °C for 45 s and extension at 72 °C for 1 min, with a final extension at 72 °C for 10 min (Torres-Garcia et al. 2023; Guerra-Mateo et al. 2025). Additionally, PCR amplification of the *BenA*, *CaM* and *RPB*2 regions in *Talaromyces* was carried out with an initial denaturation at 94 °C for 5 min, followed by 35 cycles of denaturation at 94 °C for 45 s, annealing at 55 °C for 45 s (*BenA* and *CaM*) or 52 °C (*RPB*2) and extension at 72 °C for 1 min, with a final extension at 72 °C for 10 min (Visagie et al. 2014; Pyrri et al. 2021). The PCR products were visualised on 1% agarose gels stained with ethidium bromide under UV light, purified using the NucleoSpin® Gel and PCR Clean-up Kit (Macherey-Nagel, Germany) and sent to 1^st^ BASE (Kembangan, Malaysia) for sequencing.

### Phylogenetic analyses

Both forward and reverse reads of ITS, LSU, *BenA*, *CaM* and *RPB*2 sequence data were automatically assembled via Sequencher 5.4.6 (Nishimura 2000). The sequences generated in this study were subjected to a BLAST search tool via NCBI website to exclude contamination and identify closely-related families and genera (www.ncbi.nlm.nih.gov/blast; accessed 15 December 2025). Closely-related sequences to our taxa were retrieved from GenBank based on BLAST similarity and recent publications as presented in Tables 1, 2. Sequence alignments were generated with MAFFT v. 7 (http://mafft.cbrc.jp/alignment/server) (Katoh et al. 2019) and final improvements were manually edited in BioEdit v.7.0.5.2 (Hall 2004). Sequence alignments were performed on full-length loci, including both exons and introns for protein-coding genes. Following alignment, each locus was processed via TrimAl v.1.2 (Capella-Gutiérrez et al. 2009) with a gap threshold (-gt) of 0.2 to eliminate poorly-aligned regions. Subsequently, final improvements to single-locus alignments and the combination of multi-locus sequence datasets were performed using BioEdit v.7.0.5.2. The alignment of concatenated datasets in FASTA format was converted to PHYLIP and NEXUS formats presented by using ALTER (Alignment Transformation Environment online, http://sing.ei.uvigo.es/ALTER/) (Glez-Peña et al. 2010). Phylogenetic relationships of the new species were assessed through Maximum Likelihood (ML) and Bayesian Inference (BI) analyses. Both RAxML and Bayesian analyses were performed under the online tool CIPRES Science Gateway portal (Miller et al. 2010).

**Table 1.** Taxa, strain numbers and corresponding GenBank accession numbers of taxa used in the phylogenetic analysis of *Malbranchea* in this study.

Fungal taxa	Strain number	GenBank accession numbers			
		ITS	LSU	*BenA*	*RPB*2
*Auxarthronopsis bandhavgarhensis*	CBS 134524^T^	HQ164436	JQ048938	OM047599	-
*A. guizhouensis*	LC5705^T^	KU746668	KU746714	KU746759	KY883229
*Malbranchea albolutea*	CBS 125.77^T^	MH861039	KT155094	OM047591	-
*M. aurantiaca*	CBS 127.77^T^	KT155769	AB040704	-	-
*M. californiensis*	CBS 129.62^T^	AF038352	AY176711	-	-
*M. cavernosa*	URM 8445^T^	ON862930	ON862923	OP672389	OP290517
*M. cavernosa*	URM 8534	ON862931	ON862924	OP672390	-
*M. cavernicola*	SDBR-CMU738^T^	PX868695	PX884884	PX896329	PX896333
*M. cavernicola*	SDBR-CMU739	PX868696	PX884885	PX896330	PX896334
*M. chinensis*	FMR 18267	ON720190	ON720729	OP425706	OP425715
*M. chinensis*	CGMCC3.19572^T^	MK329076	MK328981	MK336102	-
*M. chlamydospora*	AC0702 IV	KJ413382	-	KJ413354	-
*M. chlamydospora*	RV 24809^T^	AJ271425	-	-	-
*M. chrysosporioidea*	CBS 128.77^T^	AB361632	AB359413	-	-
*M. circinata*	CBS 129.77^T^	KT155623	MZ435921	OM047594	-
*M. compacta*	CBS 200.64^T^	AJ271574	AB040692	OM047592	-
*M. concentrica*	CBS 112861^T^	AJ271428	-	-	-
*M. conjugata*	CBS 247.58^T^	HF545313	AB075325	HE974414	HE974413
*M. conjugata*	UAMH 3874	KC470855	-	-	-
*M. dendritica*	CBS 131.77^T^	AY177310	AB359416	OM047595	-
*M. echinulata*	FMR 17906^T^	ON720198	ON720737	OP425705	-
*M. filamentosa*	CBS 581.82^T^	AY177298	AB359417	-	-
*M. filamentosa*	UAMH 9987	AY177301	-	-	-
*M. flava*	CBS 132.77^T^	AB361633	AB359418	-	-
*M. flocciformis*	CBS 133.77^T^	AB361634	AB359420	OM047596	-
*M. fulva*	CBS 135.77^T^	KT155630	AB359422	OM047597	-
*M. graminicola*	CBS 582.82^T^	-	AB359423	-	-
*M. guangxiensis*	LC12465	MK329081	-	MK336107	-
*M. guangxiensis*	CGMCC 3.19634^T^	MK329080	MK328985	MK336106	-
*M. gymnoascoides*	CBS 146930^T^	LR701757	LR701758	-	-
*M. gymnoascoides*	SDBR-CMU870	PX868697	PX884886	PX896331	PX896335
*M. gymnoascoides*	SDBR-CMU871	PX868698	PX884887	PX896332	PX896336
*M. irregularis*	FMR 19016^T^	ON720191	ON720730	OP425710	OP425719
*M. kuehnii*	CBS 539.72^T^	AJ271417	AB040691	-	-
*M. longispora*	CBS 135817^T^	HG326873	HG326874	-	-
*M. multiseptata*	CBS 146931^T^	LR701759	LR701760	-	-
*M. ostraviensis*	CBS 132919^T^	HE974452	-	HE974417	HE974411
*M. ostraviensis*	FMR 18693	ON720199	ON720738	OP425707	OP425716
*M. parafilamentosa*	FMR 20151^T^	PP344599	PQ849178	PQ891872	PV498623
*M. parafilamentosa*	IHEM 28255	OU989280	OU641135	PQ891873	PV498624
*M. phuphaphetensis*	TBRC 162521^T^	OP856532	OP856522	OQ144969	-
*M. pseudauxarthron*	IFO 31701^T^	AJ271572	-	-	-
*M. pseudoreticulata*	UAMH 3117^T^	AJ426452	-	-	-
*M. pulchella*	CBS 202.38^T^	AB361638	AB359426	-	-
*M. reticulata*	FMR 18696	ON721310	ON720783	-	-
*M. sedimenticola*	FMR 19564^T^	PP344596	PQ849174	PQ891868	PV498620
*M. sedimenticola*	FMR 20150	PP344597	PQ849175	PQ891869	-
*M. seminuda*	FMR 19403^T^	PP344598	PQ849177	PQ891871	-
*M. sexualis*	FMR 20852^T^	PQ859458	PQ849179	PQ891874	-
*M. sinuata*	FMR 18266^T^	ON720195	ON720734	OP425704	OP425714
*M. thaxteri*	CBS 248.58^T^	AY177305	-	HE974416	HE974412
*M. umbrina*	CBS 105.09^T^	AY177309	MH866116	HE974415	HE974407
*M. zuffiana*	CBS 219.58^T^	AY177306	AY176712	OM047593	-

Note: superscript “^T^” denotes ex-type strain. Newly-generated sequences are formatted in bold. “-”: no data available in GenBank.

**Table 2.** Taxa, strain numbers and corresponding GenBank accession numbers of taxa used in the phylogenetic analysis of *Talaromyces* sect. *Trachyspermi* ser*. Miniolutei* in this study.

Fungal taxa	Strain number	Series	GenBank accession numbers			
			ITS	*BenA*	*CaM*	*RPB*2
*Talaromyces africanus*	CBS 147340^T^	* Miniolutei *	OK339610	OK338782	OK338808	OK338833
*T. calidominioluteus*	CBS 113167	* Miniolutei *	OK339611	OK338785	OK338816	OK338836
*T. calidominioluteus*	CBS 147313^T^	* Miniolutei *	OK339612	OK338786	OK338817	OK338837
*T. chongqingensis*	CBS 147316	* Miniolutei *	OK339609	OK338781	OK338807	OK338832
*T. chongqingensis*	CGMCC 3.20482^T^	* Miniolutei *	MZ358001	MZ361343	MZ361350	MZ361357
*T. gaditanus*	CBS 996.72	* Miniolutei *	MH860641	OK338774	OK338813	OK338826
*T. gaditanus*	CBS 169.81^T^	* Miniolutei *	MH861318	OK338775	OK338802	OK338827
*T. germanicus*	CBS 147314^T^	* Miniolutei *	OK339619	OK338799	OK338812	OK338845
*T. minioluteus*	CBS 642.68^T^	* Miniolutei *	JN899346	MN969409	KJ885273	JF417443
*T. minnesotensis*	CBS 142381^T^	* Miniolutei *	LT558966	LT559083	LT795604	LT795605
*T. minnesotensis*	DTO 055-D3	* Miniolutei *	OK339617	OK338795	OK338810	OK338854
*T. pangmaphaensis*	SDBR-CMU740^T^	* Miniolutei *	PX861517	PX870625	PX870627	PX870623
*T. pangmaphaensis*	SDBR-CMU741	* Miniolutei *	PX861518	PX870626	PX870628	PX870624
*T. samsonii*	CBS 137.84^T^	* Miniolutei *	MH861709	OK338798	OK338824	OK338844
*T. samsonii*	CBS 147356	* Miniolutei *	OK339598	OK338777	OK338804	OK338829
*T. udagawae*	CBS 579.72^T^	* Miniolutei *	JN899350	-	KX961260	MN969148
*T. xishuangbannaensis*	CGMCC 3.28743^T^	* Miniolutei *	PV085761	PV102711	PV102722	PV102731
*T. xishuangbannaensis*	XCW_SN 529	* Miniolutei *	PV085763	PV102713	PV102723	PV102733
*Talaromyces* sp.	CBS 147336	* Miniolutei *	OK339608	OK338780	OK338814	OK338846
*T. trachyspermus*	CBS 373.48^T^	* Trachyspermi *	MH856401	-	KJ885281	JF417432
*T. trachyspermus*	CBS 116556	* Trachyspermi *	KM066170	KM066126	MK451694	-

Note: superscript “^T^” denotes ex-type strain. Newly-generated sequences are formatted in bold. “-”: no data available in GenBank.

ML analysis was carried out with RAxML-HPC v.8 on ACCESS (8.2.12), using the default settings, but adapted with the following: rapid bootstrap analysis type, the GTRGAMMA model and 1,000 bootstrap support replicates (Felsenstein 1985; Stamatakis 2006). BI analysis was performed using MrBayes v.3.0b4 (Huelsenbeck and Ronquist 2001), with the best-fit model of sequence evolution determined by MrModelTest 2.2 (Nylander 2004), based on the Akaike Information Criterion (AIC). The posterior probabilities (Rannala and Yang 1996; Zhaxybayeva and Gogarten 2002) were determined by the following Markov Chain Monte Carlo sampling (MCMC). Six simultaneous Markov chains were run for 1,000,000 generations, with trees sampled every 100^th^ generation. The pre-burn was set to 5 and the run was automatically stopped when the mean standard deviation of the split frequency reached below 0.01 (Maharachchikumbura et al. 2015). Phylogenetic trees were visualised and exported in EMF. format via Figtree v. 1.4.0 (http://tree.bio.ed.ac.uksoftware/figtree/) (Rambaut 2006). ML bootstrap values (MLBP), equal to or greater than 50% and Bayesian posterior probabilities (BIPP) equal to or greater than 0.95, are indicated on the resulting tree topology. The final phylogram was edited and prepared using Adobe Photoshop CC 2018 software.

### Genealogical concordance phylogenetic species recognition analyses

Phylogenetically closely-related species were analysed using the Genealogical Concordance Phylogenetic Species Recognition (GCPSR) model by performing a pairwise homoplasy index (PHI) test (Bruen et al. 2006), as implemented in SplitsTree v.4 (Huson and Bryant 2006; Quaedvlieg et al. 2014), to determine the recombination level within phylogenetically closely-related species. A PHI index below 0.05 threshold (Φw < 0.05) indicates significant recombination present in the dataset. The relationship between closely-related species was visualised by constructing split graphs from the concatenated datasets using the LogDet transformation and splits decomposition options in SplitsTree v.4.

## Results

### Fungal strains

In this study, six fungal strains (SB7, RM007, SO65, SO66, SC1 and RM001) were identified and deposited under the accession numbers SDBR-CMU738, SDBR-CMU739, SDBR-CMU740, SDBR-CMU741, SDBR-CMU870 and SDBR-CMU871, respectively. Based on morphological characteristics, four strains (SDBR-CMU738, SDBR-CMU739, SDBR-CMU870 and SDBR-CMU871) were assigned to *Malbranchea*, whereas the other two strains (SDBR-CMU740 and SDBR-CMU741) were assigned to *Talaromyces*. Subsequently, a multi-locus phylogenetic approach was employed to confirm their phylogenetic placement.

### Phylogenetic analyses

#### Phylogenetic analyses of *Malbranchea*

**Figure 1.**
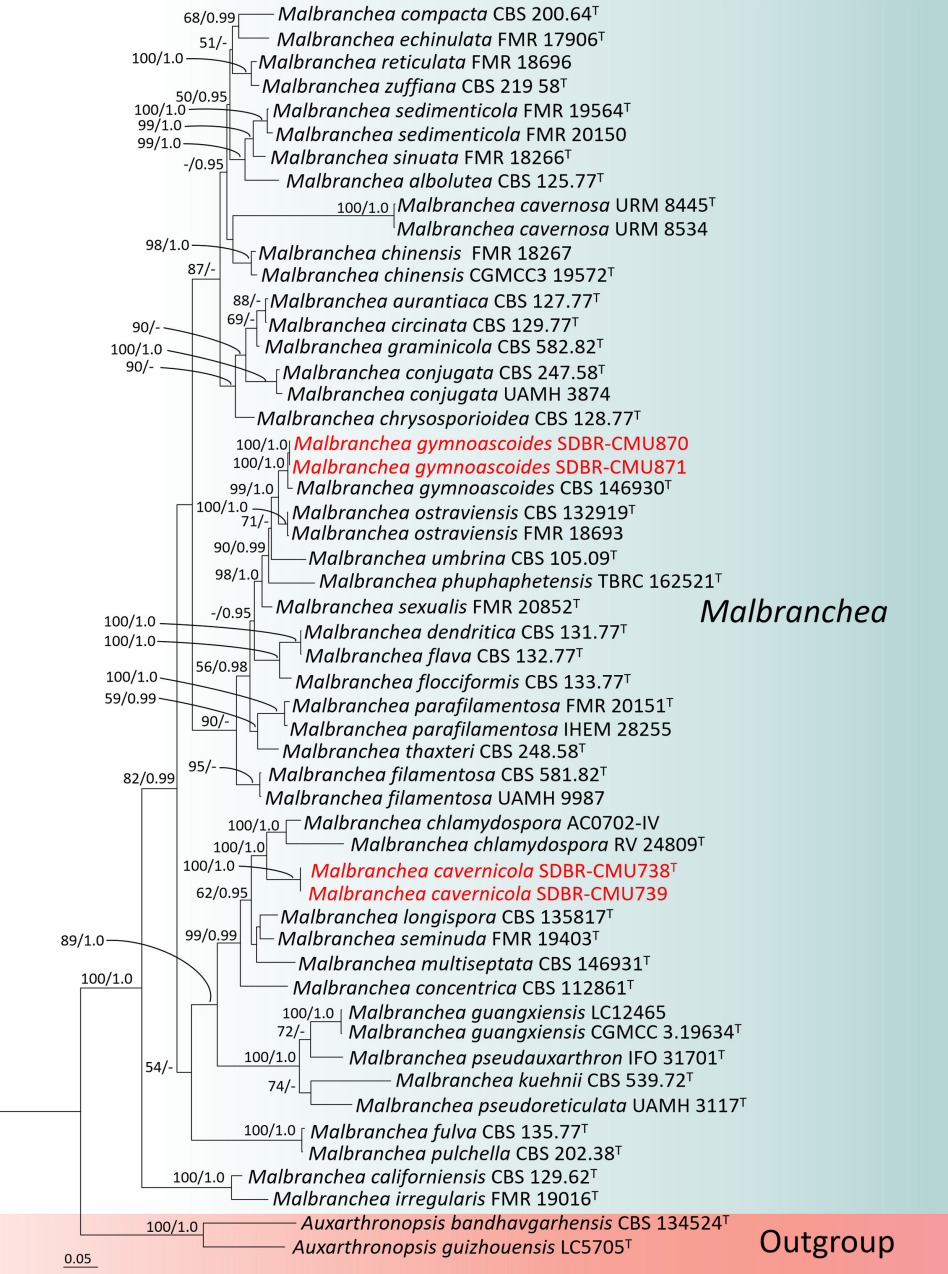
The ML phylogenetic tree, based on the combined dataset of ITS, LSU, *BenA* and *RPB*2 sequences. *Auxarthronopsis bandhavgarhensis* (CBS 134524) and *A. guizhouensis* (LC5705) were set as outgroup. Nodes were annotated if the Maximum Likelihood bootstrap support value was ≥ 50% (MLBP, left) or if the Bayesian posterior probability was ≥ 0.95 (BIPP, right). The newly-generated sequences are indicated in red bold. The ex-type strains are indicated with superscript “^T^”.

The concatenated ITS, LSU, *BenA* and *RPB*2 dataset contained 53 strains, which comprised 2957 characters (ITS 1–669 bp, LSU 670–1528 bp, *BenA* 1529–2055 bp, *RPB*2 2056–2957 bp, including gaps). *Auxarthronopsis bandhavgarhensis* CBS 134524 (ex-type) and *A. guizhouensis* LC5705 (ex-type) were used as the outgroup taxa. The best-scoring RAxML tree with a final likelihood value of -19161.362602 is presented. The matrix had 1248 distinct alignment patterns, with 46.99% of undetermined characters or gaps. Estimated base frequencies were as follows: A = 0.224005, C = 0.260314, G = 0.288599, T = 0.227081; substitution rates AC = 0.990545, AG = 2.615003, AT = 1.432928, CG = 0.590309, CT = 4.619356, GT = 1.000000; gamma distribution shape parameter α = 0.267201. The best-fit models used for the BI analysis were GTR+I+G for ITS and LSU, SYM+G for *BenA* and SYM+I+G for *RPB*2. The topologies of the phylogenetic trees obtained from ML and BI analyses were similar. Therefore, a phylogenetic tree generated from the ML analysis is shown in Fig. 1. Our phylogenetic tree shows the same topologies generally consistent with the recent study of Guerra-Mateo et al. (2025). Phylogenetic analysis placed four strains (SDBR-CMU738, SDBR-CMU739, SDBR-CMU870 and SDBR-CMU871) within the genus *Malbranchea*. Two strains, SDBR-CMU738 and SDBR-CMU739, formed a distinct monophyletic lineage with 100% MLBS and 1.0 BIPP support and sister taxon to *M. chlamydospora* [RV 24809 (ex-type) and AC0702 IV]. These two strains are hereby described as a new species, *M. cavernicola*. The remaining two strains, SDBR-CMU870 and SDBR-CMU871, clustered with *M. gymnoascoides* CBS 146930 (ex-type) in a well-supported clade (100% MLBP, 1.0 BIPP) and were, therefore, identified as *M. gymnoascoides*.

#### Phylogenetic analyses of *Talaromyces* sect. *Trachyspermi* ser. *Miniolutei*

**Figure 2.**
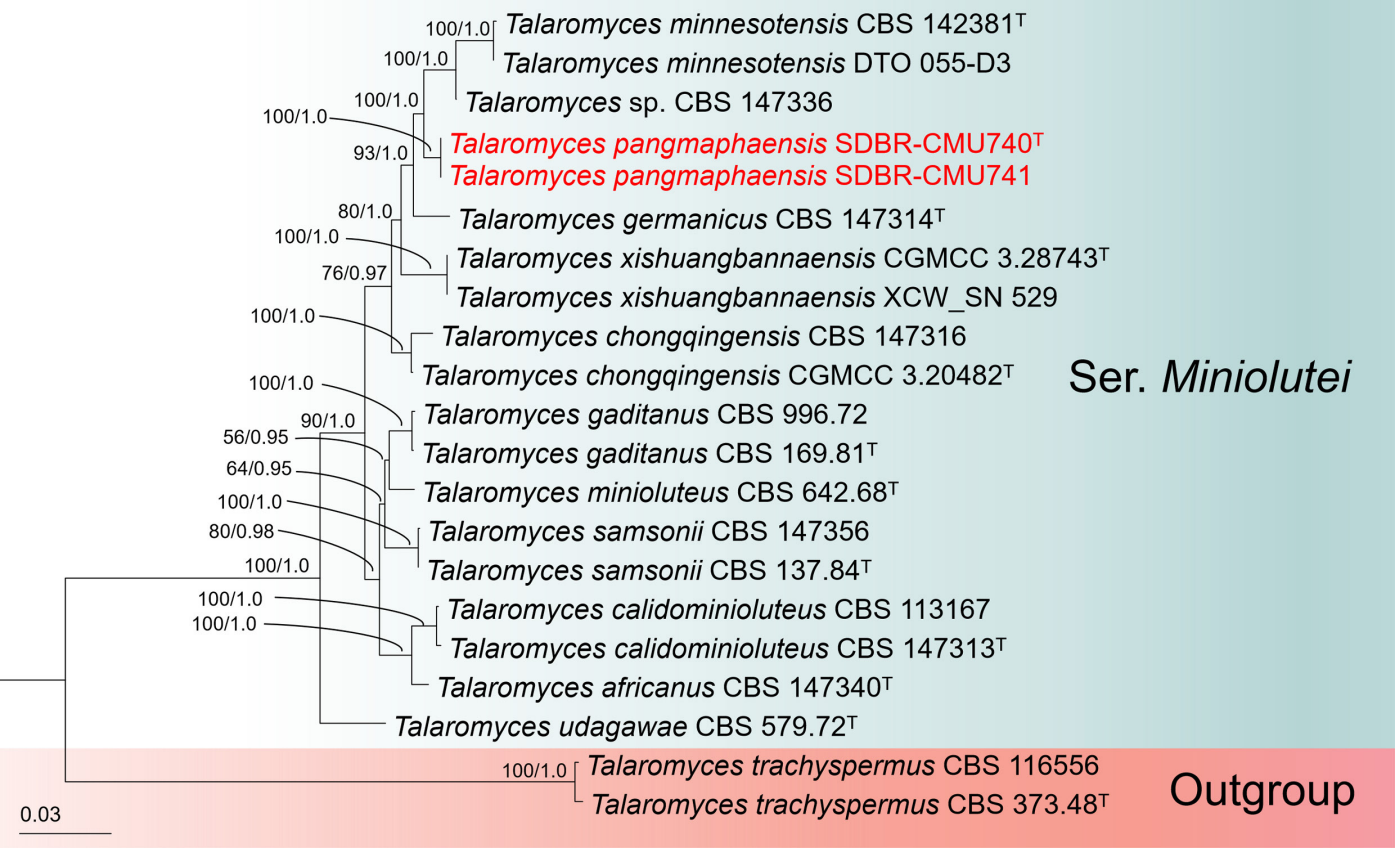
The ML phylogenetic tree, based on combined dataset of *BenA*, *CaM*, *RPB*2 and ITS sequences. *Talaromyces trachyspermus* (CBS 373.48, CBS 116556) was set as outgroup. Nodes were annotated if the Maximum Likelihood bootstrap support value was ≥ 50% (MLBP, left) or if the Bayesian posterior probability was ≥ 0.95 (BIPP, right). The newly-generated sequences are indicated in red bold. The ex-type strains are indicated with superscript “^T^”.

The concatenated *BenA*, *CaM*, *RPB*2 and ITS dataset contained 21 strains, which comprised 2610 characters (*BenA* 1–484 bp, *CaM* 485–1021 bp, *RPB*2 1022–2042 bp, ITS 2043–2610 bp, including gaps). *Talaromyces trachyspermus* CBS 373.48 (ex-type) and CBS 116556 were used as the outgroup taxon. The best-scoring RAxML tree with a final likelihood value of -7272.972337 is presented. The matrix had 441 distinct alignment patterns, with 9.88% of undetermined characters or gaps. Estimated base frequencies were as follows: A = 0.260730, C = 0.260345, G = 0.260932, T = 0.236497; substitution rates AC = 2.149835, AG = 3.515984, AT = 1.592356, CG = 0.735677, CT = 6.928853, GT = 1.000000; gamma distribution shape parameter α = 0.176781. The best-fit models used for the BI analysis were SYM+G for *BenA* and *CaM*, SYM+I for *RBP*2 and GTR+I+G for ITS. The topologies of the phylogenetic trees obtained from ML and BI analyses were similar. Therefore, a phylogenetic tree generated from the ML analysis is shown in Fig. 2. Our phylogenetic tree shows similar topologies to the previous studies (Pyrri et al. 2021; Peng et al. 2025). Phylogenetic analysis placed strains SDBR-CMU740 and SDBR-CMU741 within *Talaromyces* ser. *Miniolutei*, forming a distinct monophyletic clade supported by strong statistical evidence (100% MLBP, 1.0 BIPP). They formed a sister clade to *Talaromyces* sp. CBS 147336 (undescribed species) and *T. minnesotensis* (CBS 142381, ex-type; DTO 055-D3). A previous study demonstrated that *Talaromyces* sp. CBS 147336 is distinct from *T. minnesotensis*, based on morphological and multi-locus phylogenetic analyses, remains undescribed and may represent a novel taxon (Pyrri et al. 2021). Our results support the species delimitation framework proposed by Houbraken et al. (2020) and Pyrri et al. (2021), which recommends the use of multiple loci (*BenA*, *CaM* and *RPB*2) for the recognition of new *Talaromyces* species, rather than reliance on a single locus. Accordingly, *T. pangmaphaensis* is introduced as a new species.

#### PHI test results

**Figure 3.**
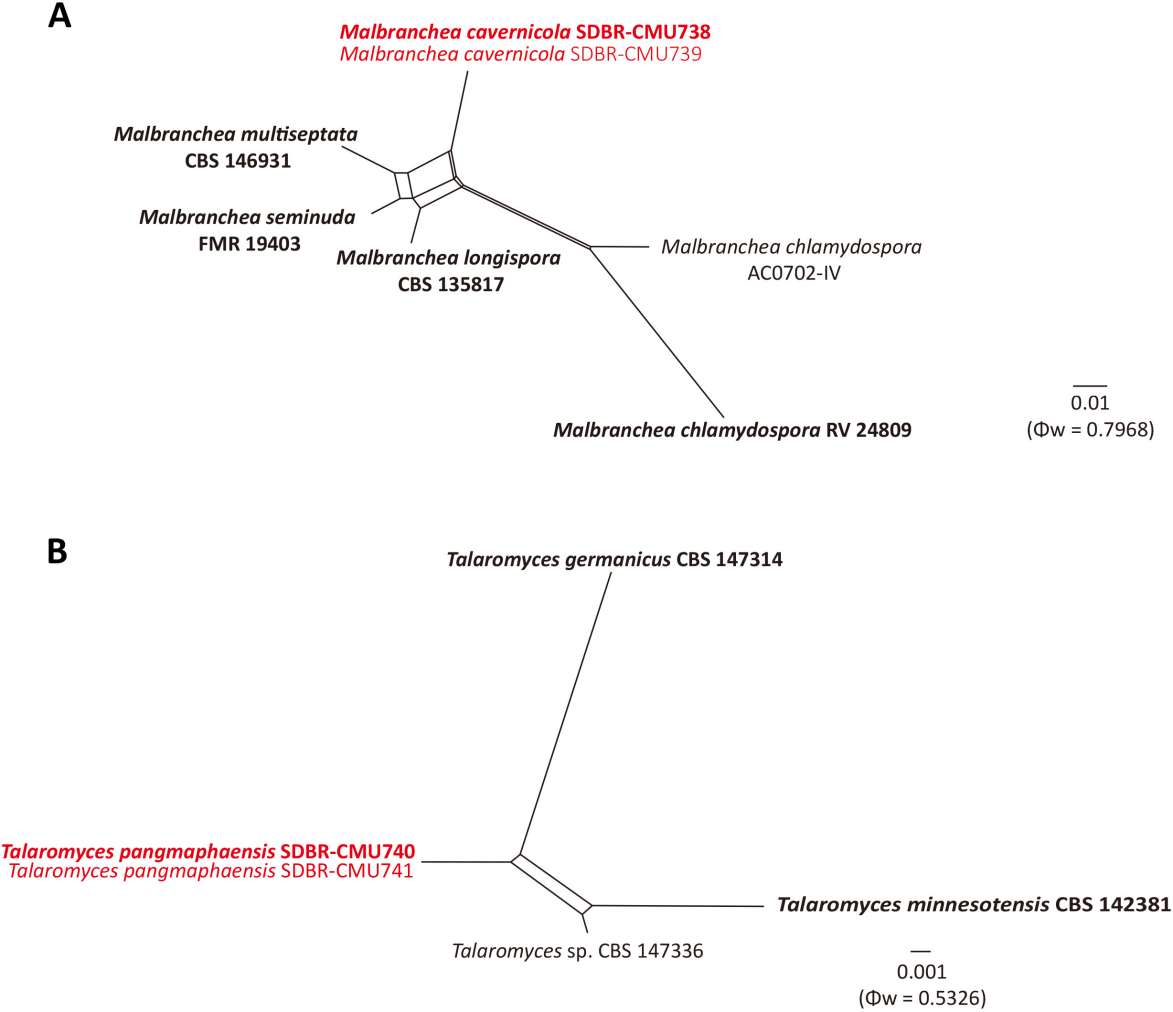
Split graphs showing the results of the pairwise homoplasy index (PHI) test of *Malbranchea cavernicola* (**A**) and *Talaromyces pangmaphaensis* (**B**) with closely-related taxa using LogDet transformation and splits decomposition. A PHI test result with Φw < 0.05 indicates significant recombination amongst the isolates included in the alignment. The new taxa are in red and each PHI test values and scale bars are given in the bottom right corner. The ex-type strains are indicated in bold.

A PHI test was conducted to assess recombination levels between the newly-described species and their phylogenetically closely-related taxa. A four-locus PHI test using ITS, LSU, *BenA* and *RPB*2 was conducted for *M. cavernicola* and closely-related species, including *M. multiseptata* CBS 146931 (ex-type), *M. seminuda* FMR 19403 (ex-type), *M. longispora* CBS 135817 (ex-type) and *M. chlamydospora* AC0702-IV (ex-type) and RV 24809. The PHI test yielded Φw = 0.7968 (Fig. 3A). For *T. pangmaphaensis*, a four-locus dataset (*BenA*, *CaM*, *RPB*2 and ITS) was analysed with *T. germanicus* CBS 147314 (ex-type), *T. minnesotensis* CBS 142381 (ex-type) and *Talaromyces* sp. CBS 147336 and the PHI test yielded Φw = 0.5326 (Fig. 3B). Therefore, PHI tests of *M. cavernicola* and *T. pangmaphaensis* indicated no significant recombination events between the newly-described taxa and their phylogenetically closely-related species (Φw > 0.05), supporting their recognition as distinct species.

### Taxonomy

#### Malbranchea cavernicola

Taxon classificationFungiOnygenalesOnygenaceae

G .Q. Zhang, J. Kumla, & N. Suwannar. sp. nov.

B12E6106-0E6F-596B-9DE6-1284E6212ACE

862365

Fig. 4

##### Etymology.

*cavernicola*, referring to the cavernicolous habitat where the soil sample containing the new fungus was collected.

**Holotype**. Thailand • Mae Hong Son Province, Pang Mapha District, Phi Man Long Long Rak Cave, 19°33'57"N, 98°10'44"E, elevation 701 m, 7 April 2024, isolated from soil, Jaturong Kumla & Nakarin Suwannarach, CMUB40078 (preserved in a metabolically inactive state as dried specimen), living culture (ex-type) SB7 = SDBR-CMU738, GenBank numbers: PX868695 (ITS), PX884884 (LSU), PX896329 (*BenA*), and PX896333 (*RPB*2).

##### Description.

Mycelium immersed and superficial, composed of hyaline, septate, branched, smooth-walled, 2.0–3.0 µm wide hyphae. Racquet hyphae present. Anamorph with well-developed fertile hyphae, arising laterally from vegetative hyphae, branched and forming randomly intercalary and terminally arthroconidia, 1.0–4.0 µm wide. Arthroconidia enteroarthric, aseptate, hyaline, smooth- and thin-walled, subcylindrical to cylindrical or Y-shaped, sometimes barrel-shaped, 3.5–30.0 × 1.0–3.5 µm (*x̄* = 14.5 × 2.2 µm; *n* = 40), secession rhexolytic. Chlamydospores and teleomorph not observed.

##### Culture characteristics

**(4 weeks at 25 °C)**. Colonies on PDA reaching 66–67 mm in diameter, circular, concentrically zonate, slightly elevated, margins entire; sporulation moderate; colony dense, cottony, white to yellowish-white (1A2) at the centre, pastel yellow (1A4) towards margins; reverse greenish-yellow (1A6) at margins; exudates absent; soluble pigments absent. On PCA, reaching 30–50 mm in diameter, circular, margins irregular; aerial mycelia extremely sparse, sporulation moderate; colony greyish-orange (5B4) at centre and orange-white (5A2) towards margins; reverse white at margins; exudates absent; soluble pigments absent. On OA, reaching 68–70 mm in diameter, circular, smooth with indistinct radial furrows, margins entire; sporulation moderate; colony flattened, granulose, white (4A1) at centre, yellowishwhite towardsmargins; exudates absent; soluble pigments absent. On PYEA, reaching 63–70 mm in diameter, circular, concentrically zonate, margins entire; sporulation moderate at the centre; colony flattened, slightly fimbriate, granulose, white (1A1), reverse orange-white (5A2); exudates absent; soluble pigments absent.

**Figure 4.**
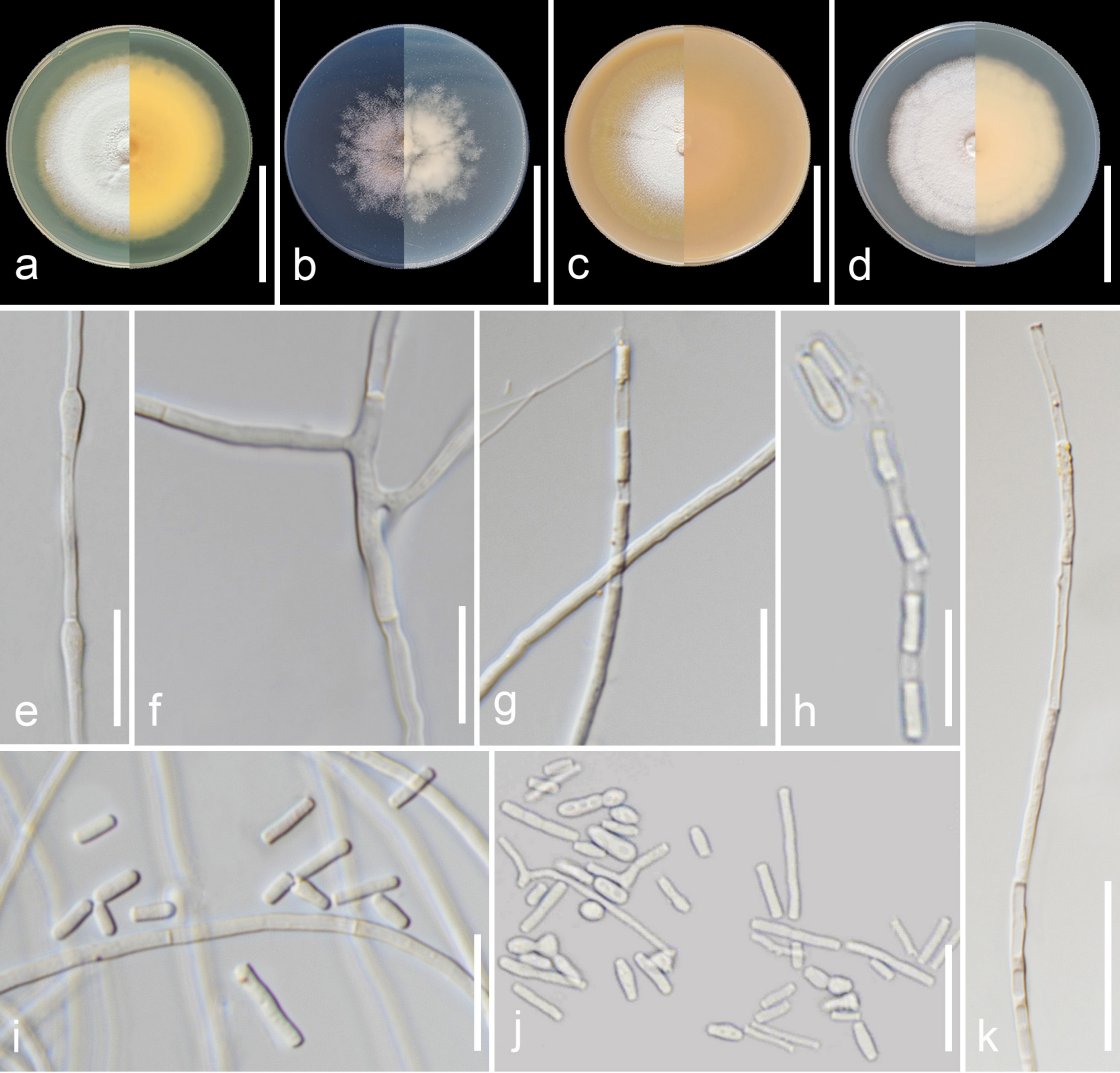
Morphological characteristics of *Malbranchea cavernicola* (SDBR-CMU738, ex-type). **a–d**. Colonies on PDA, PCA, OA and PYEA (left to right) at 25 °C for 4 weeks (right: front side; left: reverse side); **e**. Racquet hyphae; **f–h, k**. Fertile hyphae with intercalary and terminal arthroconidia; **i, j**. Arthroconidia. Scale bars: 5 cm (**a–d**); 10 μm (**e**); 15 μm (**f–j**); 30 μm (**k**).

##### Additional strain examined.

Thailand • Mae Hong Son Province, Pang Mapha District, Phi Man Long Long Rak Cave, 19°33'57"N, 98°10'44"E, elevation 701 m, 7 April 2024, isolated from soil, Jaturong Kumla & Nakarin Suwannarach, living culture RM007 = SDBR-CMU739, GenBank numbers: PX868696 (ITS), PX884885 (LSU), PX896330 (*BenA*) and PX896334 (*RPB*2).

##### Habitat and distribution.

Cave soil and known only from northern Thailand.

##### Notes.

Phylogenetically, *M. cavernicola* formed a sister clade to *M. chlamydospora* strains RV 24809 (ex-type) and AC0702 IV, with strong support (100% MLBP, 1.0 BIPP) (Fig. 1). Sequence comparisons showed that the ITS sequence shared 92.98% and 94.20% similarity with *M. chlamydospora* strains RV 24809 and AC0702 IV, respectively, while the *BenA* sequence shared 92.6% similarity to *M. chlamydospora* strain AC0702 IV. Moreover, PHI analyses revealed no evidence of significant recombination between *M. cavernicola* and *M. chlamydospora* (Fig. 3A). Morphologically, *M. cavernicola* exhibits an anamorph consistent with the generic description of *Malbranchea* (Rodríguez-Andrade et al. 2021). However, it presents racquet hyphae, lacks chlamydospores and produces longer arthroconidia (3.5–30.0 × 1.0–3.5 μm), which differ from those of *M. chlamydospora* (2.0–10.0 × 2.5–3.5 μm) (Rodríguez-Andrade et al. 2021). Based on combined morphological and phylogenetic evidence, *M. cavernicola* (SDBR-CMU738 and SDBR-CMU739) is introduced as a new species.

#### Malbranchea gymnoascoides

Taxon classificationFungiOnygenalesOnygenaceae

Rodr.-Andr., Stchigel & Cano, IMA Fungus 12: 13 (2021)

B28F4C14-8375-5E1C-915D-6FACE82C26DC

835212

Fig. 5

##### Description.

Vegetative hyphae septate, hyaline, smooth- and thin-walled, mostly straight, rarely branched, 2.0–3.0 μm wide. Racquet hyphae present. Anamorph consisting in undifferentiated fertile hyphae which form randomly intercalary and terminally arthroconidia, 1.5–3.5 μm. Arthroconidia enteroarthric, aseptate, hyaline, smooth- and thin-walled, mostly barrel-shaped, sometimes cylindrical, 3.5–15.0 × 1.5–2.5 μm (*x̄* = 7.5 × 2.0 µm; *n* = 45), detached by rhexolysis. Chlamydospores and teleomorph not observed in this study.

##### Culture characteristics

**(4 weeks at 25 °C)**. Colonies on PDA reaching 35–60 mm in diameter, circular, velvety to floccose, furrowed, radially sulcate extending from the centre to the edge were visible on the colony surface, margins entire; sporulation sparse; colony orange white (6A2) at centre, becoming light orange (6A4) towards margins, reverse orange (6A8) with light orange (6A4) ring at margins; exudates absent; pigments absent or pinkish to reddish. On PCA, reaching 40–45 mm in diameter, circular, velvety to floccose, margins irregular with fimbriate; sporulation sparse; colony greyish-orange (6B4) at centre and with orange (6A7) concentric circles, reverse deep orange (6A8), with light orange (6A5) at the margins; exudates absent; pigments absent. On OA, reaching 70–78 mm in diameter, circular, dense, cottony, velvety, furrowed, radial growth pattern, radially sulcate extending from the centre to the edge were visible on the colony surface, margins entire; sporulation sparse; the central part was raised with a dense orange grey (6B2) to deep orange (6A8) to orange (6A7) from centre to margins, reverse orange (6B6) to reddish-orange (7B8) from centre to margins; exudates absent; pigments absent. On PYEA, reaching 35–40 mm in diameter, circular, filiform, margins irregular; aerial mycelia extremely sparse, sporulation sparse; smooth with indistinct radial furrows and orange (6A6) to orange-white (6A2) at the margins; reverse medium orange (6A6) to orange-white (6A2) at margins; exudates absent; pigments absent.

**Figure 5.**
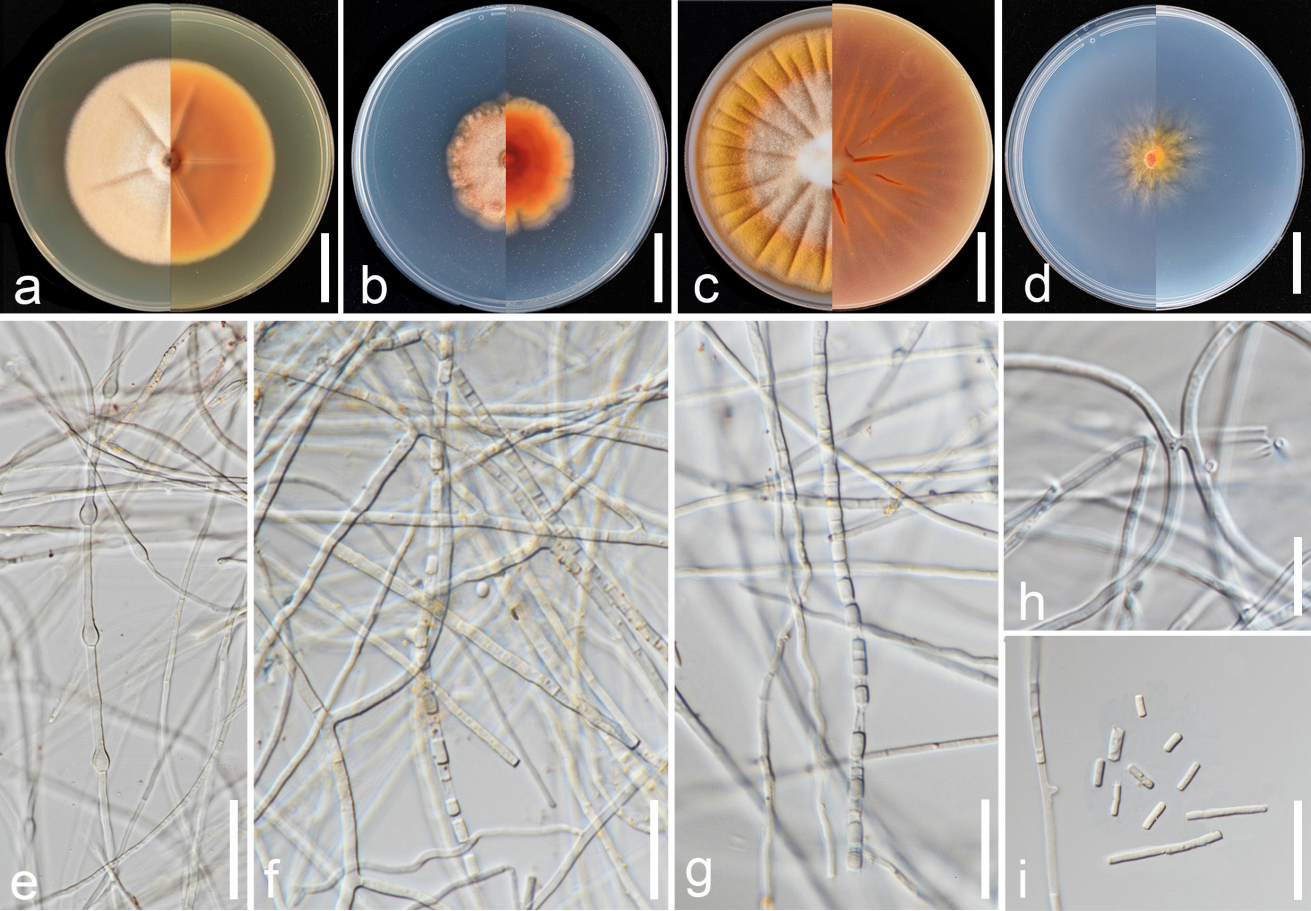
Morphological characteristics of *Malbranchea gymnoascoides* (SDBR-CMU870). **a–d**. Colonies on PDA, PCA, OA and PYEA (left to right) at 25 °C for 4 weeks (right: front side; left: reverse side); **e**. Racquet hyphae; **f, g**. Fertile hyphae with intercalary arthroconidia; **h**. Fertile hyphae with “H” shape; **i**. Arthroconidia. Scale bars: 2 cm (**a–d**); 10 μm (**e**); 15 μm (**f, g, i**); 30 μm (**h**).

##### Strain examined.

Thailand • Mae Hong Son Province, Pang Mapha District, Phi Man Long Long Rak Cave, 19°33'57"N, 98°10'44"E, elevation 701 m, 7 April 2024, isolated from soil, Jaturong Kumla & Nakarin Suwannarach, living culture SC1 = SDBR-CMU870, GenBank numbers: PX868697 (ITS), PX884886 (LSU), PX896331 (*BenA*) and PX896335 (*RPB*2); RM001 = SDBR-CMU871, GenBank numbers: PX868698 (ITS), PX884887 (LSU), PX896332 (*BenA*) and PX896336 (*RPB*2).

##### Habitat and distribution.

Human bronchial washing specimen in the USA (Rodríguez-Andrade et al. 2021); cave soil in northern Thailand (this study).

##### Notes.

Phylogenetically, strains SDBR-CMU870 and SDBR-CMU871 clustered with *M. gymnoascoides* CBS 146930 (ex-type) with strong support (100% MLBS and 1.00 BIPP; Fig. 1), forming a sister taxon to *M. ostraviensis* (CBS 132919, ex-type; FMR 18693). Sequence comparisons showed that the ITS and LSU sequences shared 99.08% and 99.89% similarity, respectively, to *M. gymnoascoides* CBS 146930 (ex-type). However, *BenA* and *RPB*2 sequence data for *M. gymnoascoides* CBS 146930 are unavailable; therefore, comparisons could not be performed. The ITS, *BenA* and *RPB*2 sequences showed 98.44%, 97.22% and 96.40% similarity, respectively, to *M. ostraviensis* CBS 132919 (ex-type). Morphologically, our strains are similar to *M. gymnoascoides* as described by Rodríguez-Andrade et al. (2021), exhibiting similar colony characteristics and arthroconidia of comparable shape, but with a broader size range (6.0–10.0 μm vs. 3.5–15.0 μm). However, chlamydospores and ascomata were not observed under the conditions of this study. In addition, *M. gymnoascoides* can be distinguished from *M. ostraviensis* by the absence of pyriform terminal conidia, chlamydospore-like cells and pinkish to red diffusible pigments on MEA and PDA (Hubka et al. 2013). Prior to this study, *M. gymnoascoides* had been recorded only in the USA (Rodríguez-Andrade et al. 2021). Therefore, strains SDBR-CMU870 and SDBR-CMU871 were identified as *M. gymnoascoides*, based on morphological and phylogenetic analyses, representing the first report of this species in Thailand.

#### Talaromyces pangmaphaensis

Taxon classificationFungiEurotialesAspergillaceae

G .Q. Zhang, J. Kumla, & N. Suwannar. sp. nov.

D83D809E-CE31-5423-967E-936177399F71

862366

Fig. 6

##### Etymology.

pangmaphaensis, referring to Pang Ma Pha District, where soil containing the new fungus was collected.

**Holotype**. Thailand • Mae Hong Son Province, Pang Mapha District, Phi Man Long Long Rak Cave, isolated from soil, 19°33'57"N, 98°10'44"E, elevation 701 m, 7 April 2024, Jaturong Kumla & Nakarin Suwannarach, CMUB40079 (preserved in a metabolically inactive state as dried specimen), living culture (ex-type) SO65 = SDBR-CMU740, GenBank numbers: PX870625 (*BenA*), PX870627 (*CaM*), PX870623 (*RPB*2), and PX861517 (ITS).

##### Description.

Sclerotia and ascomata not observed. Conidiophores biverticillate, stipes smooth-walled, non-vesiculate, 80.0–230.0 × 2.5–5.0 µm (*x̄* = 125.0 × 3.5 µm; *n* = 30). Metulae adpressed, 3–6 per stipe, 8.0–16.0 × 2.5–4.0 µm (*x̄* = 13.0 × 3.5 µm; *n* = 30); phialides acerose or ampulliform, sometimes tapering into very thin neck, 2–3 per metula, 9.0–15.5 × 1.5–3.5 µm (*x̄* = 11.5 × 2.5 µm; *n* = 30). Conidia in long, usually formed in chains at the tips of phialides, smooth, ellipsoidal to broadly ellipsoidal, with distinct connectives on both sides, 2.5–3.5 × 1.5–2.0 µm (*x̄* = 3.0 × 1.8 µm; *n* = 50).

##### Culture characteristics

**(7 days at 25 °C)**. Colonies on CYA reaching 32–35 mm in diameter, circular, slightly sunken in centre, concentrically zonate, smooth to slightly sulcate, margins entire; sporulation dense, conidia en masse greenish-grey (25D2) to greyish-orange (6B3); mycelia pastel yellow (1A4–6, 2A4); colony texture sometimes velvety, floccose; exudates present as small, clear drops at the centre; soluble pigments absent; reverse concentric rings of brownish-greyish-red (8C5) to brownish-red (8C7), deep orange (6A8–B8) at margins. On MEA, reaching 25–26 mm in diameter, subcircular to circular, margins irregular with fimbriate; sporulation moderate to strong, conidia en masse light yellow (3A4) to pale yellow (3A3); mycelia greyish-yellow (3B4) to dull yellow (3B3); colony texture floccose; exudates absent; soluble pigments absent; reverse dull yellow. On YESA, reaching 30–32 mm in diameter, subcircular to circular, concentrically zonate, smooth, margins entire; sporulation dense, conidia en masse greyish-green (25D4–E4–5); mycelia yellowish-grey (3B2) to dull yellow (3B3); colony texture floccose; exudates present as clear droplets; soluble pigments present, in greyish-yellow (4B6) shades; reverse concentric rings of brownish-orange (7C8) to reddish-orange (7B6–8), pale orange (6A3) at margins. On OA, reaching 30–32 mm in diameter, circular, slightly sunken in centre, concentrically zonate, smooth to slightly sulcate, margins entire; sporulation dense, conidia en masse dark green (25F5); mycelia pastel yellow (1A4–6, 2A4); colony texture floccose; exudates absent; soluble pigments absent; reverse concentric rings of brownish-orange (5C6–8) to light brown (6D8), then reddish-orange (7B6–8), brownish-orange at margins. On PDA, reaching 35 mm in diameter, circular, concentrically zonate, smooth to slightly sulcate, margins entire; sporulation dense, conidia en masse greyish-brown (7D3), pale red (7A3) to pastel red (7A4–5); mycelia light orange (5A5); texture floccose; exudates present as small, clear drops at the centre; soluble pigments absent; reverse concentric rings of greyish-red (8C5) to brownish-red (8C8), brownish-yellow (5C7) at margins.

**Figure 6.**
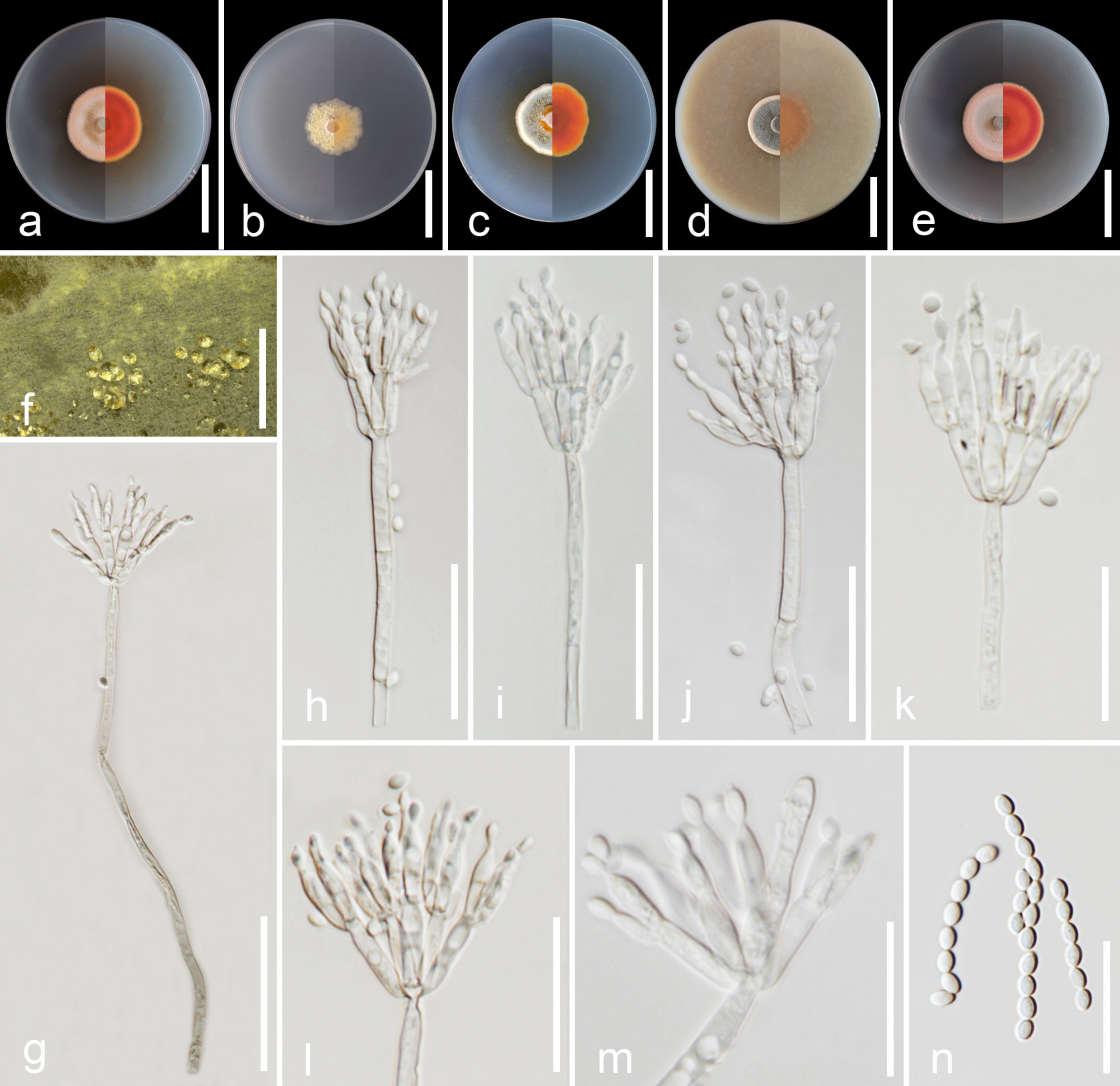
Morphological characteristics of *Talaromyces pangmaphaensis* (SDBR-CMU740, ex-type). **a–e**. Colonies on CYA, MEA, YESA, OA and PDA (left to right) at 25 °C for 7 days (right: front side; left: reverse side); **f**. Detail of colony on YESA shows clear exudates; **g–m**. Conidiophores and conidia; **n**. Conidia chains and conidia. Scale bars: 3 cm (**a–e**); 0.5 cm (**f**); 20 μm (**g–n**).

##### Additional strain examined.

Thailand • Mae Hong Son Province, Pang Mapha District, Phi Man Long Long Rak Cave, 19°33'57"N, 98°10'44"E, elevation 701 m, 7 April 2024, isolated from soil, Jaturong Kumla & Nakarin Suwannarach, living culture SO66 = SDBR-CMU741, GenBank number: PX870626 (*BenA*), PX870628 (*CaM*), PX870624 (*RPB*2), and PX861518 (ITS).

##### Habitat and distribution.

Cave soil and known only from northern Thailand.

##### Notes.

Phylogenetically, *T. pangmaphaensis* belonged to *Talaromyces* sect. *Trachyspermi*, ser. *Miniolutei* (Fig. 2) and formed a distinct monophyletic clade with strong support (100% MLBS and 1.00 BIPP). It formed a sister clade to *Talaromyces* sp. CBS 147336 (undescribed species) and *T. minnesotensis* (CBS 142381, ex-type; DTO 055-D3). PHI test results revealed no significant recombination amongst these taxa (Fig. 3B). Pairwise comparisons of *BenA*, *CaM* and *RPB*2 sequences revealed that the new species differs from *Talaromyces* sp. CBS 147336 by 3.4% (15/441 bp, including gaps), 3.1% (13/424 bp, including gaps) and 0.5% (6/1121 bp, including gaps), respectively and from *T. minnesotensis* by 3.2% (14/432 bp, including gaps) in *BenA*, 9.2% (39/424 bp, including gaps) in *CaM* and 0.8% (9/1121 bp, including gaps) in *RPB*2.

The morphological differences between *T. pangmaphaensis* and *T. minnesotensis* are provided in Table 3. Although *Talaromyces pangmaphaensis* exhibits micromorphological similarities to *T. minnesotensis*, the two species are readily distinguished by differences in conidial size (typically shorter in *T. pangmaphaensis*) and colony morphology. *Talaromyces pangmaphaensis* differs from *T. minnesotensis* by its significantly faster growth on CYA, YESA and OA, the clearly different conidia mass colour on CYA and MEA and having 2–3 phialides per metula (Table 3). In addition, *T. pangmaphaensis* produces clear exudates and greyish-yellow soluble pigments on YESA, whereas *T. minnesotensis* produces golden-red exudates and red pigments on YESA and red to deep red pigments on MEA (Pyrri et al. 2021). *Talaromyces pangmaphaensis* also differs from *Talaromyces* sp. CBS 147336 by its significantly faster growth on CYA, MEA, YESA and OA; notably, *Talaromyces* sp. CBS 147336 grows more slowly than *T. minnesotensis* (Pyrri et al. 2021). However, detailed micromorphological characteristics of *Talaromyces* sp. CBS 147336 remain undescribed; therefore, we cannot perform a direct morphological comparison with *T. pangmaphaensis*. *Talaromyces* sp. CBS 147336 was isolated from a lemon solution in Germany, whereas *T. minnesotensis* was isolated from a human ear in the USA. Consequently, *T. pangmaphaensis*, represented by strains SDBR-CMU740 and SDBR-CMU741, is introduced as a novel species belonging to *Talaromyces* sect. *Trachyspermi*, ser. *Miniolutei*, based on both morphological and phylogenetic evidence.

**Table 3.** Morphological comparison and isolation sources of *Talaromyces pangmaphaensis* and *T. minnesotensis*.

Characteristics	Fungal species	
	*Talaromyces minnesotensis*	*Talaromyces pangmaphaensis*
Colony diameter (mm) at 25 °C for 7 days / conidial mass colour		
CYA	20–24 mm / dull green to dark green	32–35 mm / greenish-grey to greyish-orange
MEA	28–30 mm / dull green to greyish-green	25–26 mm / light yellow to pale yellow
YESA	19–28 mm / greyish-green to greenish-grey	30–32 mm/ greyish-green
OA	18–22 mm / dull green to greyish-green	25–26 mm / dark green
Micromorphological characteristics		
Conidiophores (µm)	100.0–250.0 × 2.0–3.0 µm	80.0–230.0 × 2.5–5.0 µm
Metulae character / size (µm)	3–5 per stipe, 9.5–15.5 × 2.5–3.5 µm	3–6 per stipe, 8.0–16.0 × 2.5–4.0 µm
Phialides character / size (µm)	Acerose, 3–5 per metula / 11.0–15.5 × 2.0–3.0 µm	Acerose or ampulliform, 2–3 per metula / 9.0–15.5 × 1.5–3.5 µm
Conidia shape / size (µm)	Ellipsoid / 2.5–4.5 × 1.5–2.5 µm	Ellipsoid to broadly ellipsoid / 2.5–3.5 × 1.5–2.0 µm
Isolation source / country	Human ear / USA	Soil from cave / Thailand
Reference	Pyrri et al. (2021)	This study

## Discussion

Research on cave fungal diversity has increased globally over the past decade, particularly in Asia, substantially advancing fungal diversity knowledge and new taxa discovery (Jiang et al. 2017; Zhang et al. 2017, 2021; Wang et al. 2018; Chen et al. 2021; Liu et al. 2021; Kim et al. 2023). In Thailand, research on cave fungal diversity has been reported from the southern region. Cultured cave fungi isolated from five substrate types (air, water, rock, soil/sediment and organic debris) collected from Le Stegodon and Phu Pha Phet Caves in the Satun UNESCO Global Geopark, southern Thailand, revealed 319 strains representing 135 genera, based on ITS sequence data, with the most common genera being *Aspergillus* P. Micheli ex Haller, *Penicillium* Link, *Cladosporium* Link, *Talaromyces*, *Xylaria* Hill ex Schrank and *Trichoderma* Pers. (Suetrong et al. 2023). Two new species of *Talaromyces*, namely *T. phuphaphetensis* Nuankaew, Chuaseehar. & Somrith. and *T. satunensis* Nuankaew, Chuaseehar. & Somrith., were isolated from soils in Phu Pha Phet cave (Nuankaew et al. 2022). In addition, eight new fungal species were reported from Phu Pha Phet Cave, six of which were isolated from soils (*Actinomortierella caverna* C. Srihom, Preedanon, S. Saengkaewsuk & Somrith, *Hypoxylon phuphaphetense* C. Srihom, Preedanon, S. Saengkaewsuk & Somrith., *Leptobacillium latisporum* C. Srihom, Preedanon, S. Saengkaewsuk & Somrith., *Malbranchea phuphaphetensis* C. Srihom, Preedanon, S. Saengkaewsuk & Somrith., *Scedosporium satunense* C. Srihom, Preedanon, S. Saengkaewsuk & Somrith. and *Thelonectria satunensis* C. Srihom, Preedanon, S. Saengkaewsuk & Somrith.), while two species were recovered from shells of dead snails (*Sesquicillium cavernum* C. Srihom, Preedanon, S. Saengkaewsuk & Somrith. and *Umbelopsis satunensis* C. Srihom, Preedanon, S. Saengkaewsuk & Somrith.) (Preedanon et al. 2023). However, research on cave fungal diversity remains limited in northern Thailand and many other regions of the country.

Cave soil ecology is characterised by oligotrophic conditions, limited primary productivity and the absence of light, resulting in low nutrient availability (Ritz and Young 2004; Vanderwolf et al. 2013). Organic matter in cave soils is primarily derived from allochthonous inputs, such as bat guano, animal carcasses, plant debris and keratinous materials, including hair, skin and feathers (Out et al. 2016; Reis et al. 2026). These inputs serve as key sources of carbon and nitrogen, sustaining microbial communities within the cave ecosystem and supporting the growth and persistence of specialised fungi (Vanderwolf et al. 2013; Maphanga et al. 2025). Consequently, cave soils represent unique ecological niches that harbour distinct and often understudied fungal communities (Vanderwolf et al. 2013; Zhang et al. 2017, 2021; Ogórek et al. 2022; Suetrong et al. 2023; Poli et al. 2024). In this study, six fungal strains were isolated from soil samples collected within Phi Man Long Long Rak Cave, located in northern Thailand. Four fungal strains belonged to genus *Malbranchea*, while the remaining two strains belong to *Talaromyces*. Species identification of *Malbranchea* followed Guerra-Mateo et al. (2025) using combined morphological characteristics and multi-locus phylogenetic analyses (ITS, LSU, *BenA* and *RPB*2), whereas *Talaromyces* identification followed Houbraken et al. (2020), Pyrri et al. (2021) and Peng et al. (2025) using *BenA*, *CaM*, *RPB*2 and ITS. Two strains, SDBR-CMU738 and SDBR-CMU739, formed a distinct lineage within *Malbranchea*, whereas two other strains, SDBR-CMU870 and SDBR-CMU871, clustered with *M. gymnoascoides*. In addition, two strains, SDBR-CMU740 and SDBR-CMU741, formed a distinct lineage within *Talaromyces* sect. *Trachyspermi* ser. *Miniolutei*. Comparative morphological analyses with their closest phylogenetic relatives confirmed that strains SDBR-CMU738, SDBR-CMU739, SDBR-CMU740 and SDBR-CMU741 represent novel species, as detailed above. Therefore, two novel species, *M. cavernicola* (SDBR-CMU738 and SDBR-CMU739) and *T. pangmaphaensis* (SDBR-CMU740 and SDBR-CMU741), were described and species delimitation validated using GCPSR criteria by PHI tests. Furthermore, *M. gymnoascoides* (SDBR-CMU870 and SDBR-CMU871) was identified, representing the first documentation of this species in Thailand. This study can stimulate further research on the distribution and ecology of cave fungi in Thailand, Asia and globally. Furthermore, these novel fungal strains may represent promising resources for biotechnology, as cave-adapted fungi are frequently sources of unique enzymes and secondary metabolites with industrial, agricultural and pharmaceutical potential that warrant further investigation. Future investigations in Thailand should encompass multiple regions and diverse substrates within cave environments.

## Supplementary Material

XML Treatment for Malbranchea cavernicola

XML Treatment for Malbranchea gymnoascoides

XML Treatment for Talaromyces pangmaphaensis

## Additional information

### Artificial Intelligence (AI) use

The authors accept full responsibility for the content of the manuscript, including the disclosure of any use of AI.

No AI tools were used in the preparation of this manuscript.

### Author ORCIDs

G.-Q. Zhang https://orcid.org/0000-0001-5354-0607

N. Suwannarach https://orcid.org/0000-0002-2653-1913

T. Disayathanoowat https://orcid.org/0000-0002-6416-2266

R. Shoocongdej https://orcid.org/0000-0003-3018-5088

V. Domethong https://orcid.org/0009-0000-8107-5335

D.-Q. Dai https://orcid.org/0000-0001-8935-8807

R.-M. Liu https://orcid.org/0009-0009-7314-2951

N.N. Wijayawardene https://orcid.org/0000-0003-0522-5498

J. Kumla https://orcid.org/0000-0002-3673-6541

### Data availability

The datasets presented in this study can be found in online repositories. The names of the repository/repositories and accession number(s) can be found below: https://www.ncbi.nlm.nih.gov/, ITS: PX861517, PX861518, PX868695, PX868696, PX868697, PX868698; LSU: PX884884, PX884885, PX884886, PX884887; *RPB*2: PX870623, PX870624, PX896333, PX896334, PX896335, PX896336; *BenA*: PX870625, PX870626, PX896329, PX896330, PX896331, PX896332; *CaM*: PX870627, PX870628.
